# Can Psychic Depression Cause Death?

**Published:** 1914-06

**Authors:** 


### Can Psychic Depression Cause Death?

This question has been put to us recently in several ways, as: Can a person die of a nervous breakdown—referring to a case of which we have no personal knowledge, and described as apparently in good health a few days before death and with no organic disease claimed by attending physicians. Did —— die because he was discouraged—referring to a case in which different attendants made such discrepant diagnoses, along lines with which we have no special experience that no conclusion could be drawn, although a comparatively recent observation of the case, professionally, along other lines, led to some scepticism as to the existence of serious organic disease. Can a person die of a broken heart? etc.

There is no doubt but that sudden and violent psychic strain may cause death, either through inhibition of cardiac and, possibly, other vital functions, or through the production of apoplexies, etc. Neither can it be questioned that the mental state may turn the balance one way or the other in serious acute illness; nor that it may depress various functions so that the potential life of the individual is shortened and the resistence to various diseases lessened to such a degree that a given organic disease is considerably increased in mortality.

But, without adequate physical cause of death, can it be determined by general nervous depression, such as grief, discouragement, shame, etc.? This is a prevalent popular notion. It is, of course, subject to corroboration by the post hoc propter hoc argument. Is it correct or incorrect? It occurs to us that this is a question deserving serious and scientific consideration and we welcome discussion.

